# Studying the Hemibond: High-Level *Ab Initio* Calculations on Complexes of Atomic Fluorine with Halogenated Organic Molecules

**DOI:** 10.1021/acs.jpca.5c05917

**Published:** 2025-11-10

**Authors:** Götz Bucher

**Affiliations:** School of Chemistry, 3526 University of Glasgow, Joseph-Black-Building, University Avenue, Glasgow G12 8QQ, United Kingdom

## Abstract

Atomic fluorine F (^2^P_3/2_) is known to form complexes with perhalogenated solvents like carbon tetrachloride or chlorofluorocarbons. Here these complexes are studied at a very high *ab initio* level employing CCSD­(T) theory. The results show that the fluorine atom undergoes a localized three-electron bonding interaction with the halogen lone pairs, resulting in a doubly occupied σ-type orbital and a half-filled σ*-type orbital, giving a bond order of one-half. Bonding strengths range from almost negligible when the halogen atom bonded to the fluorine atom also is fluorine (0.7 kcal mol^–1^ for the CF_4_ complex) to significant when the halogen is iodine (19.6 kcal mol^–1^ for bonding to the iodine atom in methyl iodide). The degree of charge transfer from the organohalogen compound to the fluorine atom calculated varies significantly from 0.57 elementary units in the case of the methyl iodide complex to exactly zero for the carbon tetrafluoride complex. For the complexes with chlorofluorocarbon molecules, a negative charge at the fluorine atom of only ca. 0.05 elementary units is calculated, indicating that these highly reactive complexes have very little charge transfer character. An analysis of the bonding situation via the theory of Atoms in Molecules (AIM) reveals bond critical points (BCPs) between the fluorine atom and the closest halogen atom in the organohalogen compound. The Laplacian of the electron density at the halogen-fluorine BCP is always positive, indicating that the complexes should be considered to be of the Lewis acid/Lewis base type.

## Graphical abstract

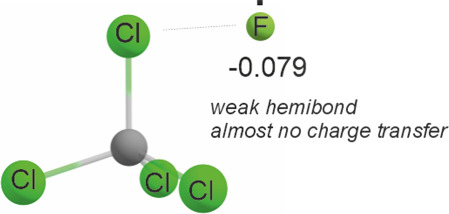

## Introduction

Atomic fluorine F (^2^P_3/2_) is a singularly reactive and unselective free radical, reacting with most substrates at the diffusion-controlled limit. Most studies on its reactivity were performed in the gas phase, where molecular F_2_ can serve as a convenient precursor.^1,2^ For a study on the reactivity of atomic fluorine in liquid condensed phase, where the use of highly hazardous molecular fluorine would be very problematic, we employed xenon difluoride^3,4^ as a precursor to atomic fluorine.^5^ Used in combination with highly unreactive solvents such as carbon tetrachloride or chlorofluorocarbons such as Freon-11 (CFCl_3_) or Freon-113 (CFCl_2_–CF_2_Cl), this enabled us to photochemically generate atomic fluorine and measure its rate constants toward a range of inorganic and organic substrates.^5^ The photolysis of XeF_2_, first investigated by Bott et al.^4^ in the gas phase, yields the XeF radical and atomic fluorine. Depending on the excitation wavelength used in the photolysis of XeF_2_, XeF can either be formed as a strongly bound exciplex or as the weakly bound ground-state XeF molecule. An excitation wavelength of λ = 204 nm represents the threshold for formation of the XeF exciplex; above this wavelength, weakly bound ground-state XeF is formed.^4^ Studying the laser flash photolysis (λ_exc_ = 248, 266, or 308 nm) of XeF_2_ employing acetonitrile as solvent, we could only detect ground-state XeF with an absorption maximum at λ = 345 nm, which decayed on a microsecond time scale according to mixed first order and second order kinetics.^5^ The use of CCl_4_, CFCl_3_, or CFCl_2_–CF_2_Cl (Freon-113) as solvent, on the other hand, resulted in the additional observation of very short-lived (τ ∼ 200 ns in Freon-113, λ_max_ = 320 nm) and intensely absorbing transients that were completely quenched by adding only minute amounts of water or compounds containing C–H bonds. Based on the extraordinary reactivity of the short-lived transients, which were quenched with rate constants at or close to the diffusion-controlled limit by almost any quencher added, we attributed them to charge-transfer complexes of atomic fluorine with the respective perhalogenated solvent. Other diagnostic reactions helping in the assignment were reactions with perfluorinated arenes (hexafluorobenzene, pentafluoropyridine) yielding perfluorinated cyclohexadienyl-type radicals exhibiting characteristic absorption around λ = 600 nm.

Our experimental work had left a few unanswered questions. For the closely related system of complexes of chlorine atoms with halogenated solvents, Chateauneuf^6,7^ had shown that the absorption maximum of the charge transfer complexes correlates with the ionization potential (IP) of the solvent, with a lower solvent IP resulting in a bathochromic shift in absorption. In our work, however, the complex of F with Freon-11 (CFCl_3_) had a shorter-wavelength absorption maximum (λ_max_ = 310 nm) than the complex of F with Freon-113 (CF_2_Cl-CFCl_2_) (λ_max_ = 320 nm), in spite of the IP of the Freon-113 (11.99 eV) being higher than the IP of Freon-11 (11.77 eV). In our 1994 paper, we argued that, possibly, the interaction was more localized for the fluorine atom complexes, implying that binding of fluorine with Freon-113 could occur at the more electron-rich −CFCl_2_ half of the molecule.

In the present contribution, the author wishes to address this issue, and to study in-depth the type of interaction found between perhalogenated solvents and halogen atoms. High-level coupled cluster (CCSD­(T)) *ab initio* theory is used to optimize the structures of complexes of fluorine atoms with small halogenated hydrocarbons, and the theory of *Atoms in Molecules* is employed to gain insight in the bonding present in these complexes.

## Computational Methods

All geometry optimizations (except DFT work) were performed using ORCA version 5.0.^8,9^ Coupled cluster theory with single and double excitations and perturbative treatment of triple excitations (CCSD­(T)) was used.^10,11^ Basis sets employed include Dunning’s aug-cc-pVTZ and aug-cc-pVQZ basis sets,^12,13^ as well as the minimally augmented ma-def2-TZVPP basis set.^14^ For compounds involving bromine or iodine atoms, a relativistic Hamiltonian (ZORA method)^15^ was used, in combination with basis sets (ZORA-ma-def2-TZVPP^16^ for carbon, hydrogen, fluorine and bromine and SARC-ZORA-TZVPP^17^ for iodine) optimized for such relativistic applications. For the analysis of the electron distribution via Bader’s Atoms in Molecules (AIM) method,^18^ the software employed was the AIMAll package.^19^ Using Gaussian16,^20^ CCSD­(T) single point energy calculations (based on the CCSD­(T) geometries optimized using ORCA) were performed. The.fchk files derived from these were then read and analyzed by AIMAll. The Gaussian single point energy jobs on systems containing bromine or iodine employed the Douglas-Kroll-Hess method^21–23^ for the relativistic Hamiltonian, and the Jorge-TZP-DKH basis set,^24^ which was taken from the Basis Set Exchange website.^25^

DFT calculations employed the M06–2X functional^26^ in combination with an aug-cc-pVTZ basis set.^12,13^ They were performed using Gaussian16.^20^

No vibrational analyses were performed at the CCSD­(T) level of theory.

## Results and Discussion

To gauge the interaction of a fluorine atom with a halogen atom bonded to carbon, first the complexes of F with methyl fluoride, methyl chloride, methyl bromide, and methyl iodide were studied, and for comparison the complex between methane and F. The complexes were optimized at the CCSD­(T) level of theory, employing aug-cc-pVQZ and aug-cc-pVTZ basis sets. In the case of methyl bromide and methyl iodide, a relativistic Hamiltonian (for optimization ZORA, in some single point energy calculations Douglas-Kroll-Hess) was employed, along with basis sets optimized for use in relativistic calculations.

Figure 1 shows the optimized geometries of complexes of F (^2^P_3/2_) with CH_3_F, CH_3_Cl, CH_3_Br, CH_3_I, and CH_4_.

**Figure 1:**
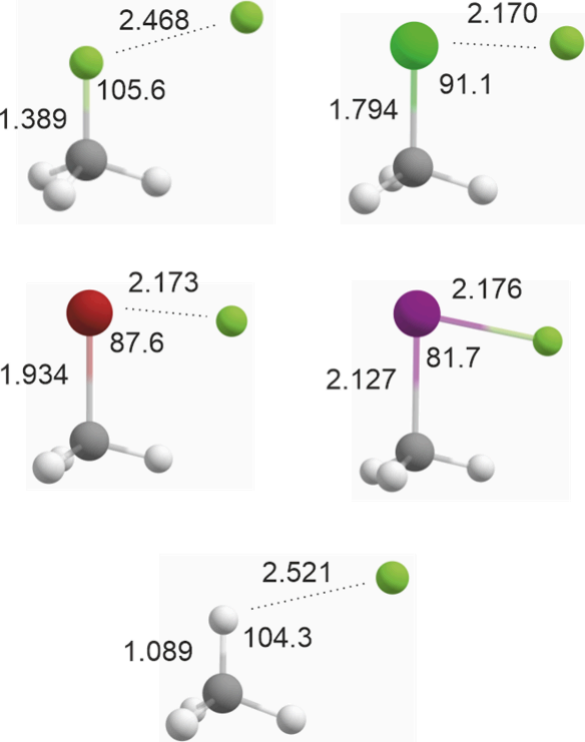
Optimized geometries of complexes of atomic fluorine with monohalomethanes and methane. Top left: F × CH_3_F (CCSD­(T)/aug-cc-pVQZ). Top right: F × CH_3_Cl (CCSD­(T)/aug-cc-pVTZ). Middle left: F × CH_3_Br (CCSD­(T)­(ZORA)/ma-ZORA-def2-TZVPP). Middle right: F × CH_3_I (CCSD­(T)­(ZORA)/ZORA-def2-TZVPP; I: SARC-ZORA-TZVPP). Bottom: F × CH_4_ (CCSD­(T)/aug-cc-pVQZ). All distances in Å, C-X-F angles in degrees.

The optimized structures shown in Figure 1 reveal some trends. First, with increasing atomic weight of the methyl halide halogen atom, the interaction between the fluorine atom and the methyl halide halogen atom becomes stronger, as evidenced by a halogen-fluorine distance that becomes increasingly shorter relative to the sum of the van-der-Waals radii of halogen (and at least going from the methyl fluoride complex to the methyl chloride complex, also in absolute terms). Second, the C-X-F angle becomes more and more acute with increasing atomic weight of the halogen atom. The C-X bond lengths, on the other hand, change only slightly with complexation of a fluorine atom. E.g., for methyl iodide as the system showing the strongest interaction, the C–I bond length is calculated to be slightly shorter (R = 2.127 Å) for the fluorine atom complex than for the uncomplexed methyl iodide molecule (R = 2.139 Å). The methane complex, finally, essentially is a very weak van-der-Waals type complex (the sum of the van-der Waals radii of hydrogen and fluorine is 255 pm, which is only very slightly longer than the H^···^F distance calculated here). A very similar geometry for this van-der-Waals complex had previously been calculated by Czakó et al.^27^

In case of the series of F atom complexes of CF_*x*_Cl_*y*_ (CF_3_Cl, CF_2_Cl_2_, CFCl_3_, CCl_4_), the strength of the F···Cl interaction correlates with the availability of the chlorine lone pairs for binding with the external fluorine atom, see Table 1. In general, the higher the fluorine content in CF_*x*_Cl_*y*_, the weaker the Cl^···^F interaction. Thus, R_Cl···F_ in CCl_4_^···^F (R = 2.271 Å) is longer than in the methyl chloride complex (R = 2.170 Å), but significantly shorter than in the CF_3_Cl complex (R = 2.492 Å). The change in Cl^···^F distance is mirrored by the electronic binding energies, where Δ*E* is significantly larger for the CCl_4_ complex (Δ*E* = −2.3 kcal mol^–1^) than for the CF_3_Cl complex (Δ*E* = −1.4 kcal mol^–1^), but much weaker than for the methyl chloride complex (Δ*E* = −5.8 kcal mol^–1^). In case of the Freon-113 (CF_2_Cl-CFCl_2_) complex, the same trend is observed in that the complex where the fluorine atom is bound to the −CF_2_Cl moiety is predicted to be weaker than the regioisomeric complex with the F atom bound to the −CFCl_2_ moiety.

**Table 1:** Selected Geometric Parameters and Electronic Binding Energies (Relative to Separate Substrate and F (^2^P_3/2_)) for Complexes Y_3_C-X···F

substrate	basis set	R (X···F) [Å]	R (C-X) [Å]	α (C-X···F) [°]	Δ*E* [kcal mol^–1^]
CH_4_	aug-cc-pVQZ	2.521	1.089	104.3	–0.7
CH_3_F (to F)	aug-cc-pVTZ	2.464	1.393	105.4	–1.5
CH_3_F (to F)	aug-cc-pVQZ	2.468	1.389	105.6	–1.4
CH_3_F (to H)	aug-cc-pVTZ	2.643	1.090	115.9	–0.4
CH_3_Cl	aug-cc-pVTZ	2.170	1.794	91.1	–5.8
CH_3_Br	ZORA-ma-def2-TZVPP	2.173	1.934	87.6	–8.9
CH_3_I	ZORA-def2-TZVPP^a^	2.176	2.127	81.7	–19.6
CCl_4_	aug-cc-pVTZ	2.271	1.794	95.4	–2.3
CFCl_3_ (F···F *antiperiplanar*)	aug-cc-pVTZ	2.335	1.789	94.2	–1.8
CFCl_3_ (F···F *gauche*)	aug-cc-pVTZ	2.319	1.786	93.6	–2.0
CF_2_Cl_2_	aug-cc-pVTZ	2.398	1.780	92.1	–1.6
CF_3_Cl	aug-cc-pVTZ	2.492	1.770	90.0	–1.4
CF_4_	aug-cc-pVTZ	2.833	1.323	97.4	–0.7
CF_2_Cl-CFCl_2_ (to −CFCl_2_)	aug-cc-pVDZ	2.494	1.793	91.1	–1.5
CF_2_Cl-CFCl_2_ (to −CF_2_Cl)	aug-cc-pVDZ	2.548	1.783	88.8	–1.4

a Basis set for iodine: SARC-ZORA-TZVPP.

An attempt to optimize (CCSD­(T)/aug-cc-pVTZ) a complex of CFCl_3_ with F in which the fluorine atom was bound to the fluorine atom of the CFCl_3_ molecule converged to a complex in which the fluorine atom was bound to a chlorine atom of CFCl_3_ instead. On the whole, F···F interactions in such complexes appear to be exceedingly weak, as evidenced by the very weak binding and long F^···^F distance in the CF_4_ complex. The trend observed for the correlation of Cl^···^F bond strength with C-X-F angles, finally, is opposite to what is observed for the CH_3_X^···^F complexes, in that the more acute angles are predicted for the complexes in which the fluorine atom is bound less strongly.

Figure 2 shows the geometries of complexes of CCl_4_, CFCl_3_ (two rotamers), CF_2_Cl_2_, CF_3_Cl, and CF_4_, with F (^2^P_3/2_).

**Figure 2:**
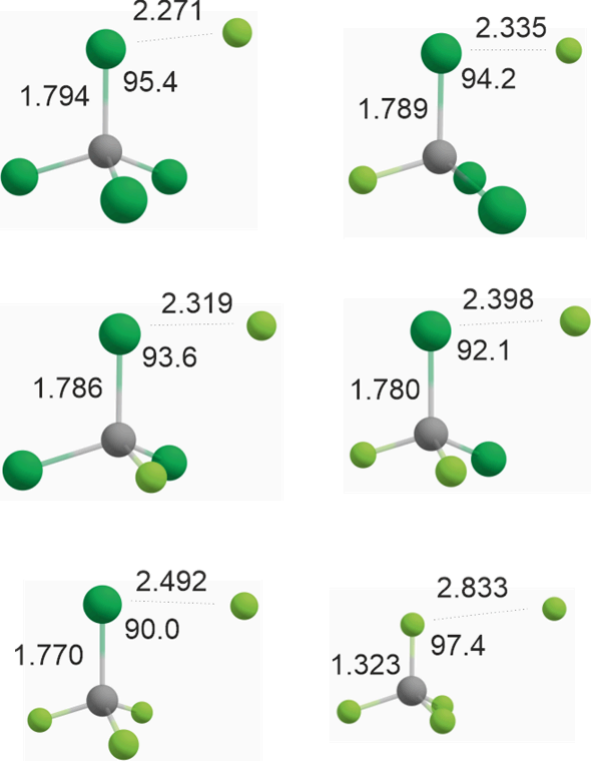
Optimized geometries (CCSD­(T)/aug-cc-pVTZ) of complexes of atomic fluorine with perhalogenated methanes. Top left: F × CCl_4_. Top right: F × CFCl_3_ (F···F *antiperiplanar*). Middle left: F × CFCl_3_ (F···F *gauche*). Middle right: F × CF_2_Cl_2_. Bottom left: F × CF_3_Cl. Bottom right: F × CF_4_. Distances in Å, angles in °. Chlorine atoms are shown in dark green, fluorine atoms in light green, carbon atoms in gray.

The strengths of the X^···^F interactions vary. The electronic energy (CCSD­(T)/aug-cc-pVQZ) of the methane – fluorine atom complex is lower than the sum of the electronic energies of methane and F (^2^P_3/2_) by Δ*E* = −0.7 kcal mol^–1^. An alternative geometry for the methyl fluoride – F complex, where the fluorine is closest to the CH_3_ moiety of methyl fluoride, has a binding strength of 0.4 kcal mol^–1^. Halogen-bonded complexes are stronger: Δ*E* CH_3_F^···^F = −1.4 kcal mol^–1^, Δ*E* CH_3_Cl^···^F = −5.8 kcal mol^–1^, and the maximum X^···^F bond strength among the compounds calculated was observed for CH_3_I^···^F with Δ*E* = −19.6 kcal mol^–1^. The variations in bonding strength observed are consistent with previous results reported in the literature on complexes of methyl halides with atomic fluorine. In work by Jacox, the reactions of fluorine atoms, matrix isolated in argon at cryogenic temperatures, with methyl chloride, -bromide and -iodide were reported.^28^ In all three cases, infrared spectra could be assigned to CH_3_Cl^···^F, CH_3_Br^···^F, and CH_3_I^···^F, respectively. In a similar study on the reaction of F atoms with methyl fluoride, no evidence had been gained for the formation of CH_3_F^···^F.^29^ The iodine compound CH_3_I^···^F had previously been observed in a gas phase crossed molecular beam experiment, and identified as an intermediate in the reaction CH_3_I + F → CH_3_ + IF.^30,31^ An analysis of the dependence of the yield of CH_3_I^···^F on the collision energy allowed Farrar and Lee to estimate the bond dissociation energy of the I–F bond in CH_3_I^···^F as 25 ± 3 kcal mol^–1^, which is slightly above the value calculated here.^30,31^ A later study on the reaction of F_2_ with a range of alkyl iodides RI (R = CH_3_, C_2_H_5_, *n*-C_3_H_7_, *i*-C_3_H_7_, *t*-C_4_H_9_, allyl) resulted in similar findings, with I–F BDEs of the adduct radicals R-I^···^F consistently measured at around 115 kJ mol^–1^ (or 27 kcal mol^–1^).^32^

Apart from binding to halogen atoms, atomic fluorine can also undergo weak binding with lone pairs present on other atoms. For example, there is computational evidence for formation of complexes like H_2_O^···^F in the reaction of F with water,^33–35^ as well with methanol,^36^ where the complex of F bound to the oxygen atom was reported to be 6.6 kcal mol^–1^ below the energies of separate CH_3_OH + F, which clearly is in a range that fits well with the results presented in Table 1.

### Orbital Interactions

Interaction of a fluorine atom with the lone pair of a carbon-bound halogen atom results in a three-electron interaction, with a doubly filled X-F σ orbital and a half filled X-F σ* orbital. Depending on the X···F distance, π-type interactions are also possible, but will be destabilizing rather than stabilizing, as they would involve doubly filled π* orbitals. This expectation is confirmed by the results of population analyses performed on the complexes investigated. As an example, Figure 3 shows the HOMO (or SOMO) and HOMO–1 to HOMO–7 of the complex of F (^2^P_3/2_) with methyl chloride (HF/aug-cc-pVTZ natural orbitals). It is clearly seen that the SOMO is σ* in character, and the HOMO–1 is σ. The orbitals below are either π*/π type (HOMO–4 and HOMO–7 involving interaction of p_*y*_ orbitals on Cl and F) or mostly localized (HOMO–2 for 2s on fluorine, HOMO–5 for 3s on chlorine). The results are consistent with previous work on the complex of atomic fluorine and water.^34^

**Figure 3:**
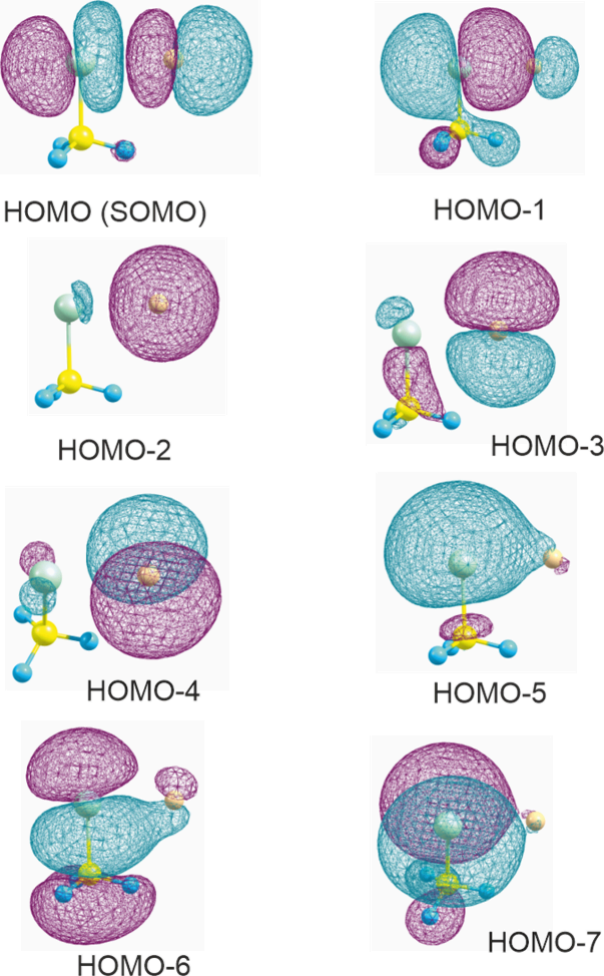
Natural orbitals of the complex of F (^2^P_3/2_) with CH_3_Cl. From a population analysis at HF/aug-cc-pVTZ, geometry CCSD­(T)/aug-cc-pVTZ. The contour value was selected as 0.015.

### Atoms in Molecules

R. Bader’s theory of Atoms in Molecules^18^ (AIM) was used to study bonding interactions in the complexes investigated. The software used to analyze the population analyses was the AIMAll software package.^19^ Table 2 lists several parameters from the AIM analyses. With the exception of methane as a substrate, for all systems studied, bond critical points (BCP) could be localized connecting the atomic F nucleus and the halogen atom to which it is bonded. In case of the F atom/methane complex, no BCP was found connecting hydrogen and fluorine. Instead, a BCP was found connecting carbon and fluorine. Table 2 lists the Laplacian of the electron density, ∇^2^ ρ, at two selected BCP – X-F and C-X. In addition, AIM charges are listed for the atomic fluorine nucleus and the halogen nucleus to which F is bound.

**Table 2:** Laplacian of the Electron Density at Selected Bond Critical Points of Complexes of F (^2^P_3/2_) with Halogenated Substrates and Methane^a^

substrate	basis set	∇^2^ρ BCP X-F	∇^2^ρ BCP C-X	charge F^b^	charge X
CH_3_F	aug-cc-pVQZ	+0.091	+0.293	–0.006	–0.758
CH_3_F	aug-cc-pVTZ	+0.088	+0.443	–0.006	–0.758
CH_3_Cl	aug-cc-pVTZ	+0.262	–0.343	–0.131	–0.242
CH_3_Br	Jorge-TZP-DKH	+0.273	–0.621	–0.326	+0.374
CH_3_I	Jorge-TZP-DKH	+0.165	–0.126	–0.572	+0.512
CH_4_	aug-cc-pVQZ	+0.040^c^	–1.152	–0.009	+0.258^c^
CCl_4_	aug-cc-pVTZ	+0.227	–0.328	–0.079	–0.097
CFCl_3_ (*anti*)	aug-cc-pVTZ	+0.200	–0.356	–0.064	–0.114
CFCl_3_ (*syn*)	aug-cc-pVTZ	+0.207	–0.360	–0.066	–0.110
CF_2_Cl_2_	aug-cc-pVTZ	+0.176	–0.389	–0.050	–0.125
CF_3_Cl	aug-cc-pVTZ	+0.143	–0.426	–0.036	–0.131
CF_4_	aug-cc-pVTZ	+0.030	–0.146	0.000	–0.754
CF_2_Cl-CFCl_2_ (to −CFCl_2_)	aug-cc-pVDZ	+0.134	–0.137	–0.042	–0.137
CF_2_Cl-CFCl_2_ (to −CF_2_Cl)	aug-cc-pVDZ	+0.115	–0.385	–0.034	–0.147

a AIM charges (in elementary units) at the atomic fluorine nucleus and at the nucleus X to which it is bonded. Single point energy population analyses (using Gaussian 16) based on geometries from ORCA optimizations as listed in Table 1.^37^

b Atomic fluorine.

c The BCP connects F with the C atom of methane. The charge listed is that at C. (Charge at H: −0.062).

The results show that the X···F interaction is always a Lewis base – Lewis acid type interaction, and never of the covalent type, as the Laplacian is always positive at the BCP connecting X and F. With the exception of the methyl fluoride complex, bonding between the carbon atom and X is covalent, as revealed by the negative values of the Laplacian. The absolute value of the Laplacian for the X···F BCP shows correlation with both the strength of the interaction (see Table 1), and with the charge at the fluorine atom (i.e., with the degree of electron transfer). The degree of electron transfer also correlates with the ionization potential of the substrate.^38^ Figure 4 shows plots of the degree of charge transfer vs the ionization potential (IP) of the complex partner, and a plot of the natural logarithm of the degree of charge transfer vs IP. In the IP range investigated, the latter is reasonably linear,^39^ with a slope of −1.32 logarithmic units per eV (R^2^ = 0.84). It is noted that in the logarithmic plot, the point for the carbon tetrafluoride complex was left out, as the calculated degree of charge transfer is exactly zero.

**Figure 4:**
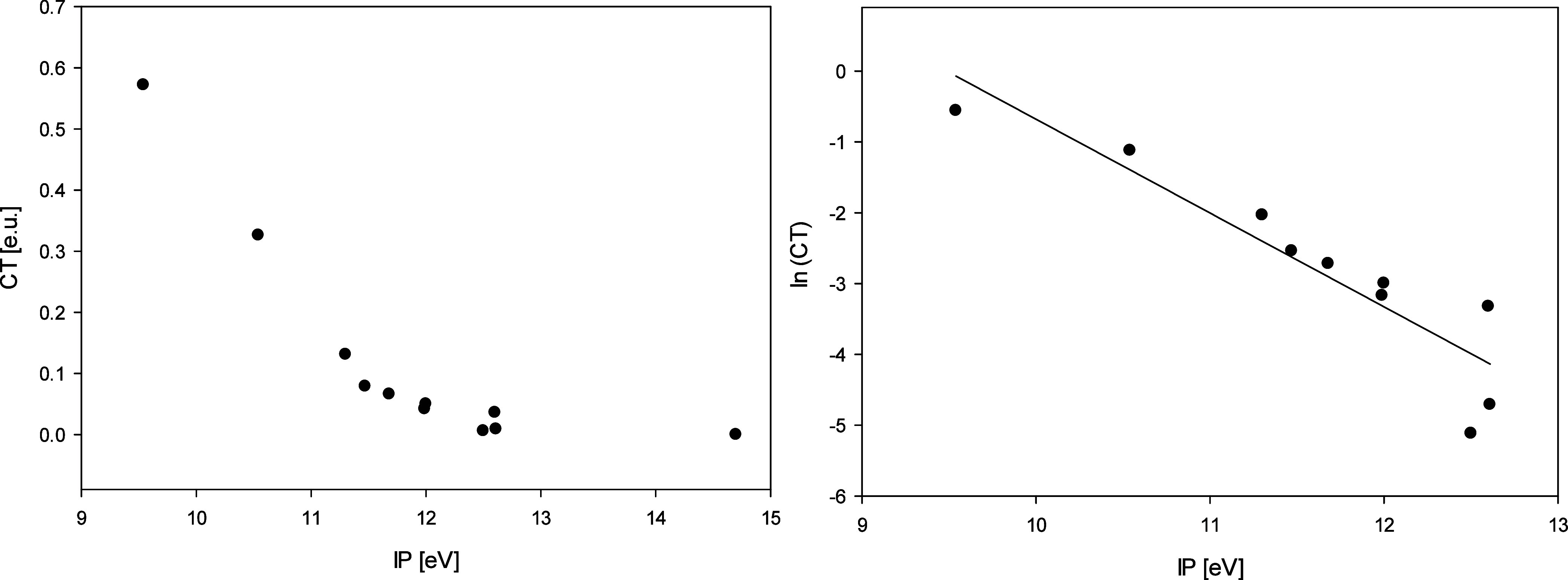
Left: plot of the calculated degree of charge transfer (CT) to the fluorine atom for a series of complexes of atomic fluorine with organohalogen compounds vs the ionization potential (IP) of the organohalogen compound. Right: plot of the natural logarithm of CT vs IP, linear regression.

Diffusion-controlled rate constants for bimolecular reactions of atomic fluorine are significantly faster (up to *k* = 1 × 10^11^ M^–1^ s^–1^ for tetrachloroethylene as quencher) than expected based on Debye’s formula.^5^ In our 1994 contribution, we attributed this to the very small size of atomic fluorine (comparable to the neon atom), which allows it to undergo diffusion through solvents faster than normal-sized organic molecules.^5^ An adduct radical of F to Freon-113, such as CF_2_Cl-CFCl_2_^···^F, however, is a normal-sized species and its diffusion coefficient should therefore not be unusually large. The fraction of fluorine atoms present as “free” atoms at any given time can be calculated from the free energy of complex formation. While vibrational frequencies (which would provide access to the entropy of complex formation) have not been calculated at the CCSD­(T) level of theory, results from DFT calculations can be used here. Using the value of the electronic binding energy of Δ*E* = −1.5 kcal mol^–1^ for CF_2_Cl-CFCl_2_^···^F as an approximation for Δ*H* and a value of Δ*S* for complex formation of −20.5 cal mol^–1^ K^–1^ (from vibrational frequencies calculated at M06–2X/aug-cc-pVTZ), we arrive at Δ*G* = +4.6 kcal mol^–1^ for the formation of CF_2_Cl-CFCl_2_^···^F from Freon-113 and F at *T* = 298 K. In other words, the unfavorable entropy term outweighs the weak enthalpy contribution, the formation of the complex is endergonic, and the atomic fluorine is so weakly bound that it behaves as if it were “free” fluorine atoms.

## Conclusion

The interaction of fluorine atoms with the lone pairs of halogen atoms can result in significant binding. This is particularly important for substrates containing bromine or iodine, but organic bound chlorine also binds atomic fluorine to a non-negligible degree. Only fluorine bound to an electron-poor carbon atom, as in CF_4_, does not interact significantly with atomic fluorine. The results presented here show that the interaction between organic bound chlorine and atomic fluorine has only a very small degree of charge transfer character. It should rather be described as a localized, but weak binding interaction, where the bonding effect of a doubly occupied Cl–F σ-orbital is largely canceled out by a half-filled Cl–F σ* orbital. The observation of non-negligible bonding between atomic fluorine and organic bound halogen also indicates that the fluorine atom (and, in analogy, other halogen atoms like atomic chlorine) could become more selective and kinetically less reactive once a substrate in a free-radical halogenation reaction contains halogen. This is likely particularly true at low temperatures, when the entropic gain associated with cleavage of the partial X-F bond does not contribute to Δ*G* as much and Δ*G* for formation of the complex would be negative.

## Supplementary Material



## Notes

Cartesian coordinates and electronic energies of all stationary points optimized. Ionization potentials used for Figure 4 (PDF)
