# Supplementary material for: Studying the Hemibond: High-Level Ab Initio Calculations on Complexes of Atomic Fluorine with Halogenated Organic Molecules
Source: J Phys Chem A. 2025 Nov 10;129(46):10655–60. doi: 10.1021/acs.jpca.5c05917 (PMC12641479; doi:10.1021/acs.jpca.5c05917)
Supplement: Supplementary file 1 [file jp5c05917_si_001.pdf]

Studying the Hemibond: High Level *ab initio* Calculations on Complexes of Atomic Fluorine with Halogenated Organic Molecules

Götz Bucher*

School of Chemistry, University of Glasgow, Joseph-Black-Building, University Avenue, Glasgow G12 8QQ, United Kingdom. goetz.bucher@glasgow.ac.uk

Supporting Information

Cartesian coordinates and electronic energies of minima calculated

Complex of atomic fluorine with methane (CCSD(T)/aug-cc-pVQZ)

C	-0.000023000	-1.259293000	0.031278000
H	0.889623000	-1.884309000	0.076353000
H	0.000031000	-0.572289000	0.876234000
H	0.000003000	-0.700582000	-0.903474000
H	-0.889750000	-1.884195000	0.076374000
F	0.000105000	1.716502000	-0.186438000

Electronic energy: -140.1057010

Methane (CCSD(T)/aug-cc-pVQZ)

C	-0.587895000	0.104646000	-1.409801000
H	-0.059237000	-0.843512000	-1.333867000
H	0.126256000	0.923761000	-1.352488000
H	-1.302046000	0.188208000	-0.592946000
H	-1.116553000	0.150121000	-2.359907000

Electronic energy: -40.4517286

Complex of atomic fluorine with methyl fluoride (bound to F) (CCSD(T)/aug-cc-pVQZ)

C	-0.000025000	-1.316708000	0.104232000
H	0.893758000	-1.938279000	0.100888000
H	0.000030000	-0.657252000	0.970308000
F	0.000012000	-0.537109000	-1.044785000
H	-0.893892000	-1.938160000	0.100911000
F	0.000106000	1.803342000	-0.261226000

Electronic energy: -239.2635732

Methyl fluoride (CCSD(T)/aug-cc-pVQZ)

C	-0.594241000	0.097360000	-1.410305000
H	-0.052932000	-0.844435000	-1.333193000
H	-1.302258000	0.192706000	-0.588382000
H	-1.115786000	0.154412000	-2.364599000
F	0.314825000	1.140051000	-1.337355000

Electronic energy: -139.6083869

Complex of atomic fluorine with methyl fluoride (bound to F) (CCSD(T)/aug-cc-pVTZ)

C	-0.000025000	-1.316290000	0.104983000
H	0.895267000	-1.938209000	0.101081000
H	0.000030000	-0.654825000	0.971315000

F	0.000012000	-0.535739000	-1.048367000
H	-0.895401000	-1.938089000	0.101104000
F	0.000106000	1.798986000	-0.259789000

Electronic energy: -239.201506

Methyl fluoride (CCSD(T)/aug-cc-pVTZ)

C	-0.594825000	0.096699000	-1.410355000
H	-0.052149000	-0.846037000	-1.333071000
H	-1.303473000	0.192758000	-0.587066000
H	-1.116703000	0.154410000	-2.366141000
F	0.316756000	1.142263000	-1.337202000

Electronic energy: -139.571297

Complex of atomic fluorine with methyl fluoride (bound to CH_3) (CCSD(T)/aug-cc-pVTZ)

C	0.650621000	0.560221000	-0.011874000
H	-0.071714000	0.316447000	-0.791451000
H	0.161884000	0.590382000	0.963115000
H	1.123750000	1.519379000	-0.224703000
F	-2.506478000	-0.290683000	0.039101000
F	1.629071000	-0.425149000	0.012021000

Electronic energy: -239.199782

Complex of atomic fluorine with methyl chloride (CCSD(T)/aug-cc-pVTZ)

C	0.000015000	-1.268861000	-0.012097000
H	0.898173000	-1.871612000	0.085966000
Cl	-0.000142000	-0.066695000	1.319237000
H	-0.000115000	-0.692858000	-0.933218000
H	-0.898169000	-1.871550000	0.086177000
F	0.000257000	1.571871000	-0.102961000

Electronic energy: -599.212475

Methyl chloride (CCSD(T)/aug-cc-pVTZ)

C	0.029852000	-0.023871000	-0.594812000
Cl	-1.347990000	-1.124936000	-0.765599000
Cl	0.536372000	0.605194000	-2.172053000
Cl	1.379200000	-0.822181000	0.230969000
H	-0.285455000	0.814066000	0.013504000

Electronic energy: -1417.841029

Complex of atomic fluorine with methyl bromide (CCSD(T)/ma-ZORA-def2-TZVPP)

C	-0.000030000	-1.426360000	0.391246000
H	0.899414000	-2.031054000	0.414719000
H	0.000034000	-0.657426000	1.156347000
Br	0.000022000	-0.442191000	-1.273351000
H	-0.899561000	-2.030924000	0.414740000
F	0.000081000	1.380754000	-0.091327000

Electronic energy: -2770.588511

Methyl bromide (CCSD(T)/ma-ZORA-def2-TZVPP)

C	-0.600378000	0.090327000	-1.410798000
H	-0.046060000	-0.838411000	-1.332598000
H	-1.296872000	0.199960000	-0.586899000
H	-1.110175000	0.161630000	-2.365240000
Br	0.670590000	1.548101000	-1.308807000

Electronic energy: -2670.7666287

Complex of atomic fluorine with methyl iodide (CCSD(T)/ZORA-def2-TZVPP; iodine: SARC-ZORA-TZVPP)

C	-0.000032000	-1.440418000	0.418936000
H	0.899484000	-2.044884000	0.449998000
H	0.000026000	-0.649690000	1.161520000
H	-0.899626000	-2.044766000	0.450015000
F	0.000089000	1.335247000	-0.053724000
I	0.000017000	-0.362690000	-1.414371000

Electronic energy: -7403.2957397

Methyl iodide (CCSD(T)/ZORA-def2-TZVPP; iodine: SARC-ZORA-TZVPP)

C	0.000000000	-0.000010000	-1.847591000
H	0.000000000	1.033628000	-2.174648000
H	-0.895158000	-0.516811000	-2.174656000
H	0.895158000	-0.516811000	-2.174656000
I	0.000000000	0.000005000	0.291513000

Electronic energy: -7303.457570

Complex of atomic fluorine with carbon tetrachloride (CCSD(T)/aug-cc-pVTZ)

C	0.289042000	0.163122000	0.384004000
Cl	0.623803000	-0.214090000	-1.337371000
Cl	-0.309527000	-1.286275000	1.195951000
Cl	1.813091000	0.688557000	1.124286000
Cl	-0.907475000	1.458308000	0.477943000
F	-1.507934000	-0.809621000	-1.844813000

Electronic energy: -1976.5974538

Carbon tetrachloride (CCSD(T)/aug-cc-pVTZ)

C	0.000000000	0.000000000	0.000000000
Cl	1.024885000	1.024885000	-1.024885000
Cl	-1.024885000	1.024885000	1.024885000
Cl	1.024885000	-1.024885000	1.024885000
Cl	-1.024885000	-1.024885000	-1.024885000

Electronic energy: -1876.965916

Complex of atomic fluorine with fluorotrichloromethane, F…F antiperiplanar (CCSD(T)/aug-cc-pVTZ)

C	0.508801000	-0.000005000	0.269355000
Cl	0.686026000	1.454302000	-0.707789000
Cl	-1.073995000	-0.000004000	1.102221000
Cl	0.686014000	-1.454301000	-0.707807000
F	1.451973000	-0.000015000	1.223800000
F	-2.309472000	0.000018000	-0.879259000

Electronic energy: -1616.608899

Fluorotrichloromethane (CCSD(T)/aug-cc-pVTZ)

C	-0.447619000	-0.012293000	0.053691000
Cl	-1.816076000	-1.119784000	-0.124182000
Cl	0.060946000	0.603627000	-1.525166000
Cl	0.900484000	-0.818206000	0.868500000
F	-0.838767000	1.027169000	0.808314000

Electronic energy: -1516.978158

Complex of atomic fluorine with fluorotrichloromethane, F…F gauche (CCSD(T)/aug-cc-pVTZ)

C	0.318693000	0.028379000	0.222703000
Cl	0.070955000	1.650254000	-0.415440000
Cl	-0.800352000	-1.127121000	-0.553791000
Cl	1.980348000	-0.512453000	-0.044361000
F	0.069627000	0.029474000	1.535643000
F	-2.695083000	-0.057378000	0.248084000

Electronic energy: -1616.609106

Complex of atomic fluorine with dichlorodifluoromethane (CCSD(T)/aug-cc-pVTZ)

C	0.527648000	-0.294147000	0.200843000
F	0.320648000	-0.204814000	1.510069000
Cl	-0.957639000	-0.884527000	-0.583120000

Cl	1.019404000	1.266779000	-0.439373000
F	1.491655000	-1.190996000	-0.007016000
F	-2.257845000	0.916873000	0.319967000

Electronic energy: -1256.624918

Dichlorodifluoromethane (CCSD(T)/aug-cc-pVTZ)

C	-0.495350000	-0.007103000	-0.787003000
F	-1.584351000	-0.624610000	-1.247101000
F	-0.417863000	1.182451000	-1.385232000
Cl	-0.647561000	0.224410000	0.956113000
Cl	0.933010000	-0.960334000	-1.194844000

Electronic energy: -1156.994568

Complex of atomic fluorine with chlorotrifluoromethane (CCSD(T)/aug-cc-pVTZ)

C	0.665935000	-0.156131000	-0.000032000
F	0.539110000	-0.912980000	-1.077608000
Cl	-0.563517000	1.117556000	-0.000056000
F	0.539283000	-0.912651000	1.077797000
F	1.873154000	0.392880000	-0.000207000
F	-2.356348000	-0.613987000	0.000108000

Electronic energy: -896.644505

Chlorotrifluoromethane (CCSD(T)/aug-cc-pVTZ)

C	-0.493040000	0.001690000	0.127920000
F	-0.499710000	-0.010094000	-1.199104000
F	-1.748299000	-0.028606000	0.557484000
F	0.127496000	-1.089878000	0.557486000
Cl	0.322430000	1.443029000	0.722717000

Electronic energy: -797.014525

Complex of atomic fluorine with carbon tetrafluoride (CCSD(T)/aug-cc-pVTZ)

C	-0.753495000	-0.063705000	-0.613640000
F	-1.025920000	0.002692000	0.676542000
F	-1.744976000	-0.672809000	-1.242918000
F	-0.607404000	1.155543000	-1.098666000
F	0.363280000	-0.746503000	-0.793725000
F	-3.796222000	1.151186000	-0.542197000

Electronic energy: -536.666072

Carbon tetrafluoride (CCSD(T)/aug-cc-pVTZ)

C	-0.039313000	-0.441248000	-0.041651000
F	-0.132269000	-0.281245000	1.266452000
F	-1.113994000	-1.068049000	-0.486106000
F	0.053643000	0.740760000	-0.624386000
F	1.035368000	-1.156449000	-0.322554000

Electronic energy: -437.03715977

Complex of atomic fluorine with 1,1,2-trichloro-trifluoroethane (bonded to Cl of $-CFCl_2$ group) (CCSD(T)/aug-cc-pVDZ)

C	0.673960000	-0.165737000	0.085226000
F	0.519258000	-0.938293000	-1.024627000
Cl	-0.575060000	1.120491000	0.062670000
F	-2.385654000	-0.574826000	-0.199381000
C	2.082713000	0.505798000	0.009173000
Cl	3.378538000	-0.697872000	-0.086749000
F	2.125449000	1.291111000	-1.096462000
Cl	0.518931000	-1.166156000	1.539357000
F	2.265731000	1.295786000	1.094338000

Electronic energy: -1853.549174

Complex of atomic fluorine with 1,1,2-trichloro-trifluoroethane (bonded to Cl of $-CFCl_2$ group) (M06-2X/aug-cc-pVTZ)

C	-0.296373000	0.087053000	0.157413000
F	-0.416424000	0.257999000	1.474684000
Cl	-1.486962000	-1.120864000	-0.369414000
F	-3.287902000	0.254461000	0.556783000
C	1.123561000	-0.478235000	-0.128968000
Cl	2.372001000	0.611599000	0.458815000
F	1.242580000	-1.652780000	0.488458000
Cl	-0.545726000	1.608510000	-0.673576000
F	1.269364000	-0.675242000	-1.435448000

Electronic energy: -1856.1165593
This stationary point has 0 vibrational modes with imaginary frequency.

Complex of atomic fluorine with 1,1,2-trichloro-trifluoroethane (bonded to Cl of $-CF_2Cl$ group) (CCSD(T)/aug-cc-pVDZ)

C	0.670916000	-0.108786000	0.014484000
F	0.547295000	-0.893419000	-1.080292000
Cl	-0.595527000	1.145009000	-0.032961000
F	0.509284000	-0.875705000	1.114457000
F	-2.351092000	-0.702284000	-0.023244000
C	2.097482000	0.527774000	0.018739000
Cl	3.316891000	-0.767956000	-0.051275000
Cl	2.333428000	1.545093000	1.456213000
F	2.208987000	1.299006000	-1.102889000

Electronic energy: -1853.548961

1,1,2-Trichloro-trifluoroethane (CCSD(T)/aug-cc-pVDZ)

C	0.667246000	-0.101049000	0.014263000
F	0.549193000	-0.893713000	-1.080374000
Cl	-0.600225000	1.137732000	-0.033359000
F	0.510835000	-0.875489000	1.114504000
C	2.096436000	0.529131000	0.018664000
Cl	3.314192000	-0.770336000	-0.051043000
Cl	2.337898000	1.544981000	1.456450000
F	2.213181000	1.299760000	-1.102630000

Electronic energy: -1753.996703

1,1,2-Trichloro-trifluoroethane (M06-2X/aug-cc-pVTZ)

C	0.716563000	-0.528916000	-0.224186000
F	0.797487000	-1.751548000	0.298394000
Cl	2.154502000	0.382353000	0.224165000
F	0.646753000	-0.642174000	-1.546648000
C	-0.574099000	0.151813000	0.310404000
Cl	-1.958773000	-0.869522000	-0.075936000
Cl	-0.764451000	1.754454000	-0.386234000
F	-0.464963000	0.251363000	1.640342000

Electronic energy: -1756.3787047
This stationary point has 0 vibrational modes with imaginary frequency.

Fluorine atom, electronic energies (CCSD(T)/basis set):

aug-cc-pVDZ: -99.550070
aug-cc-pVTZ: -99.627827
aug-cc-pVQZ: -99.652908
ZORA-ma-def2-TZVPP: -99.807667
ZORA-def2-TZVPP: -99.8070161

Ionization potentials used for Figure 4:

CH_4: 12.61 eV. CH_3Cl: 11.30 eV. CH_3F: 12.50 eV. CH_3Br: 10.54 eV. CH_3I: 9.54 eV. CCl_4: 11.47 eV. CCl_3F: 11.68 eV. CCl_2F_2: 12.00 eV. $CClF_3$: 12.60 eV. CF_4: 14.70 eV. Cl_2F-CF_2Cl: 11.99 eV.
